# Effect of “needle sensation” and the real-time changes in autonomic nervous system activity during acupuncture analgesia

**DOI:** 10.3389/fnins.2024.1349059

**Published:** 2024-03-15

**Authors:** Zehua Liu, Jinglei Huang, Dingshang Yan, Sha Liang, Shatong Zhao, Mengzhen Zhang, Zhongwen Li, Chuliang Jiang, Xiang Yin, Yingjun Zhang, Tianshu Hou, Min Feng

**Affiliations:** ^1^School of Rehabilitation Medicine and Healthcare, Hunan University of Medicine, Huaihua, China; ^2^Hunan Provincial Key Laboratory of Dong Medicine, Hunan University of Medicine, Huaihua, China; ^3^School of Clinical Medicine, Hunan University of Medicine, Huaihua, China; ^4^Department of Preventive Traditional Chinese Medicine, Chengdu Integrated TCM and Western Medical Hospital, Chengdu, China

**Keywords:** acupuncture analgesia, needle sensation, autonomic nervous system, heart rate variability, traditional Chinese medicine, vagal activity

## Abstract

**Introduction:**

Acupuncture analgesia (AA) is widely used in clinical practice. The autonomic nervous system (ANS) may be an important pathway for acupuncture signal transduction. However, real-time changes in autonomic function during AA and the effect of “needle sensation” remain unclear.

**Methods:**

We established a human pain model in healthy adults and randomly assigned 128 participants to the model, sham acupuncture, and acupuncture groups in a 1:1:2 ratio. Heart rate variability (HRV), including total power (TP), low-frequency power (LF), high-frequency power (HF), ratio of LF to HF (LF/HF), standard deviation of the normal-normal intervals (SDNN), and root mean square of successive interval differences (RMSSD), were used to assess autonomic function. The visual analog scale (VAS) and efficiency were used to assess the analgesic effect of acupuncture. The Massachusetts General Hospital acupuncture sensation scale (MASS) was used to indicate the intensity of the needle sensation. Anxiety levels were also measured. Finally, the correlation of MASS with HRV, VAS, and anxiety levels was analyzed.

**Results:**

VAS decreased after 10 min of needling and 5 min after needle withdrawal in the acupuncture group compared with those in the model group (*p* = 0.038, *p* = 0.020). The efficacy rates were 82.0, 50.0, and 61.3% in the acupuncture, model, and sham groups, respectively. These represent significant differences between the acupuncture group and the model and sham acupuncture groups (*p* < 0.001 in each case). No differences were observed between the model and sham acupuncture groups. HF, TP, SDNN, and RMSSD were all increased in the acupuncture group compared with those in the model group (*p* = 0.045, *p* = 0.041, *p* = 0.002, *p* = 0.006, respectively). No differences were observed in the sham acupuncture group compared to the model group (*p* = 0.632, *p* = 0.542, *p* = 0.093, *p* = 0.222, respectively). The LF and LF/HF did not differ among all three groups. A positive correlation was observed between MASS and RMSSD_2_, LF_2_, RMSSD_4_, TP_4_, VAS_5,_ and anxiety levels.

**Conclusion:**

AA was associated with enhanced vagal activity. The intensity of needle sensation was positively correlated with vagal and sympathetic nerve activities. Acupuncture is an effective means of regulating autonomic function, and needle sensation may be an important modulator.

## 1 Introduction

Acupuncture is an external treatment originating from traditional Chinese medicine (TCM), which is now used in more than 183 countries and regions worldwide (Fan et al., 2020). Acupuncture is effective for multisystem disorders and associated pain (He et al., 2020). Researchers have intensively studied the mechanism of acupuncture analgesia (AA) and found that it is related to the release of endogenous analgesic substances, increased local blood flow, and improved muscle movement synergy (Qiao et al., 2020). A growing body of research suggests that the autonomic nervous system (ANS) is involved in AA (Lin and Chen, 2008; Shi et al., 2023).

The ANS includes sympathetic, parasympathetic, and enteric branches. The vagus nerve is a cranial nerve and the main component of the parasympathetic nervous system. The sympathetic and parasympathetic nerves synergize and antagonize each other in the bidirectional regulation of autonomic functions. Studies have shown that autonomic dysfunction, characterized by decreased vagal activity and increased sympathetic activity, is present in the elderly in various chronic disorders, including pain (Forte et al., 2022). As the modulation of autonomic function may reduce the incidence of related diseases, the role of the ANS in disease and health is being increasingly emphasized (Dormal et al., 2021). A biostatistical study identified six important future research directions from current trends and hotspots in acupuncture research, including “the value of acupuncture in autonomic regulation” (Xiong et al., 2022). A recent meta-analysis showed that acupuncture can regulate the ANS mainly by decreasing sympathetic activity, elevating vagal tone, and regulating their balance; this has been observed when utilizing AA (Hamvas et al., 2023). However, the real-time changes in autonomic function during AA and the effect of “needle sensation,” which is considered a key factor in the efficacy of acupuncture, remain unclear. Answering these questions is important for studying the autonomic regulatory mechanisms of AA and exploring the objective indicators of needle sensation.

Heart rate variability (HRV) is the number of temporal variations between cardiac cycles and can be obtained by quantitatively describing the beat-to-beat variation in the R-R intervals on electrocardiogram (ECG) recordings. Different indicators of HRV may reflect activities of the various ANS components. The combined measurement of high-frequency power (HF), root mean square of successive interval differences (RMSSD), and standard deviation of the normal-normal intervals (SDNN) reflect vagal activity. Low-frequency power (LF) reflects sympathetic activity. The ratio of LF to HF (LF/HF) reflects the balance between the two, and total power (TP) reflects the overall activity of the ANS. Due to the sensitivity, objectivity, and noninvasiveness of HRV, it has become the most commonly used index for assessing autonomic activity in clinical and scientific research (Forte et al., 2022). Studies have shown that HRV is closely related to changes in immediate biochemical indicators in blood serum during pain, providing a real-time objective indicator for AA and changes in autonomic activity during AA (Chen et al., 2016). Therefore, this study established a pain model in healthy people using HRV as an indicator to observe the real-time changes in autonomic activity during AA and the role played by “needle sensation” to explore the autonomic regulatory effects in AA and its influencing factors.

## 2 Materials and methods

### 2.1 Study design

**Figure 1:**
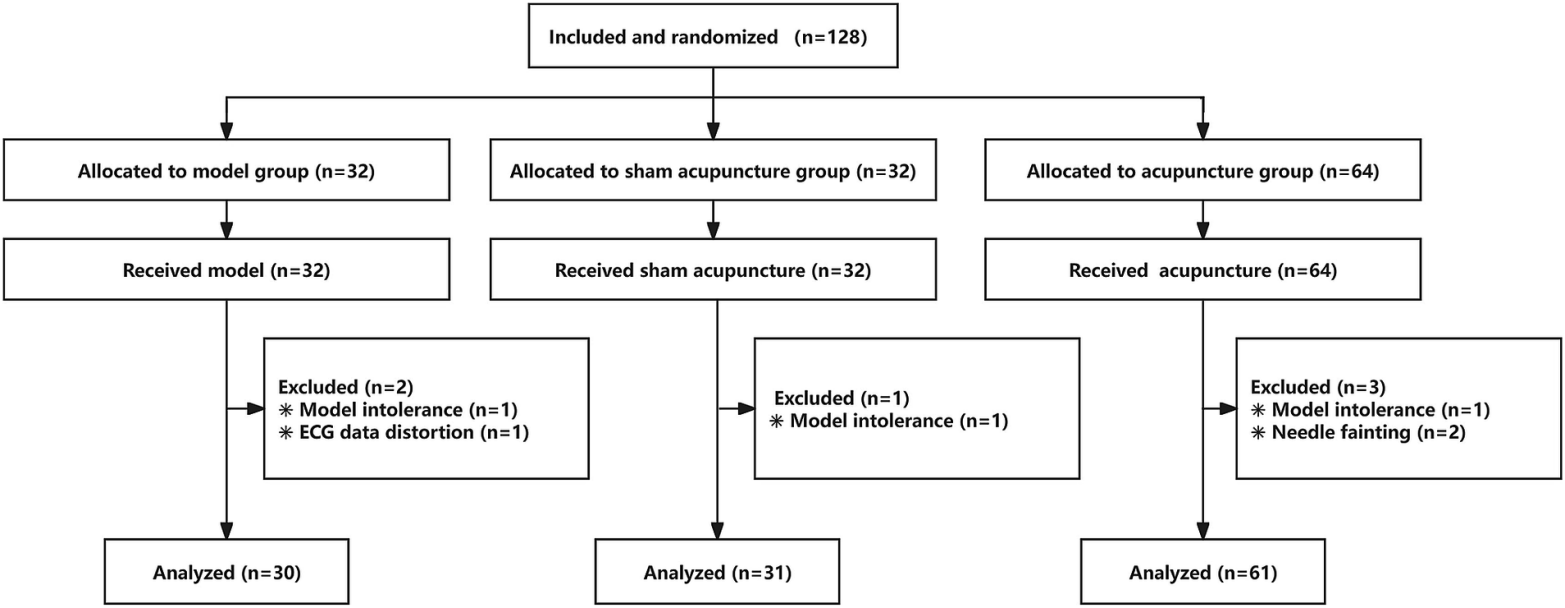
Study flow chart.

This was a randomized, controlled, single-blind study. In our previous study on acupuncture analgesia (AA) at the Hegu point, the Visual Analog Scale (VAS) scores were 3.6, 5.1, and 5.6 in the acupuncture, sham acupuncture, and model groups, respectively. For the current study, we conducted a sample size calculation considering a two-tailed test, 90% power, and a significance level of 0.05, with a dropout rate of 10% and a ratio of model group to sham acupuncture group to acupuncture group of 1:1:2. This resulted in a total of 128 participants across the three groups. Participants in the current study were recruited from the Hunan University of Medicine and randomly divided into model (no treatment control), sham acupuncture (placebo control), and acupuncture groups at a ratio of 1:1:2 using SPSS software and sealed envelopes that were coded sequentially. The operators were acupuncturists who received a 10-day standardized training in modeling and acupuncture methods, electrocardiogram recording, and information collection, and passed the assessment successfully. The operators were unaware of the participants’ enrollment until the envelopes were opened, and the intervention was administered to the participants in strict accordance with the requirements of each group. Participants were placed in a separate room with their right hand across a cloth curtain to prevent them from seeing the intervention their right hand received. The study design is depicted in Figure 1. The study was performed in accordance with the Declaration of Helsinki, and the protocol was approved by the Ethics Committee of Hunan University of Medicine (No. 2020 [H091401]). Clinical Trial Registration Number: ChiCTR2100046699.

### 2.2 Participants

The inclusion criteria were: (1) adults aged 18–25; (2) no knowledge or prior experience of acupuncture; (3) no history of heart disease, hypertension, diabetes, neurological and/or mental health conditions, chronic pain, sensory disorders, or allergy to *Capsicum* fruit; (4) no record of treatment or medication for disease within the 2 weeks prior to the study; (5) non-smokers; and (6) voluntary participation in the study by providing signed informed consent.

The exclusion criteria were: (1) pregnant women, women preparing for pregnancy, and breastfeeding women; (2) individuals with a bleeding tendency; (3) individuals who frequently consume spicy food; (4) individuals with a history of needle-related fainting; (5) individuals suspected of having any disease; and (6) any other circumstances that could confound the results of the study. Individuals with incomplete records were automatically excluded.

### 2.3 Modeling method

An acute pain model was constructed by covering the participant’s lower lip with a cotton patch (4 cm x 1 cm) impregnated with 0.5 mL of capsaicin (98.55%, Ruifensi, Chengdu, China) solution (0.1% solution configured with 30% alcohol) for 15 min (Fang et al., 2015).

### 2.4 Interventions

#### 2.4.1 Acupoint localization

**Figure 2:**
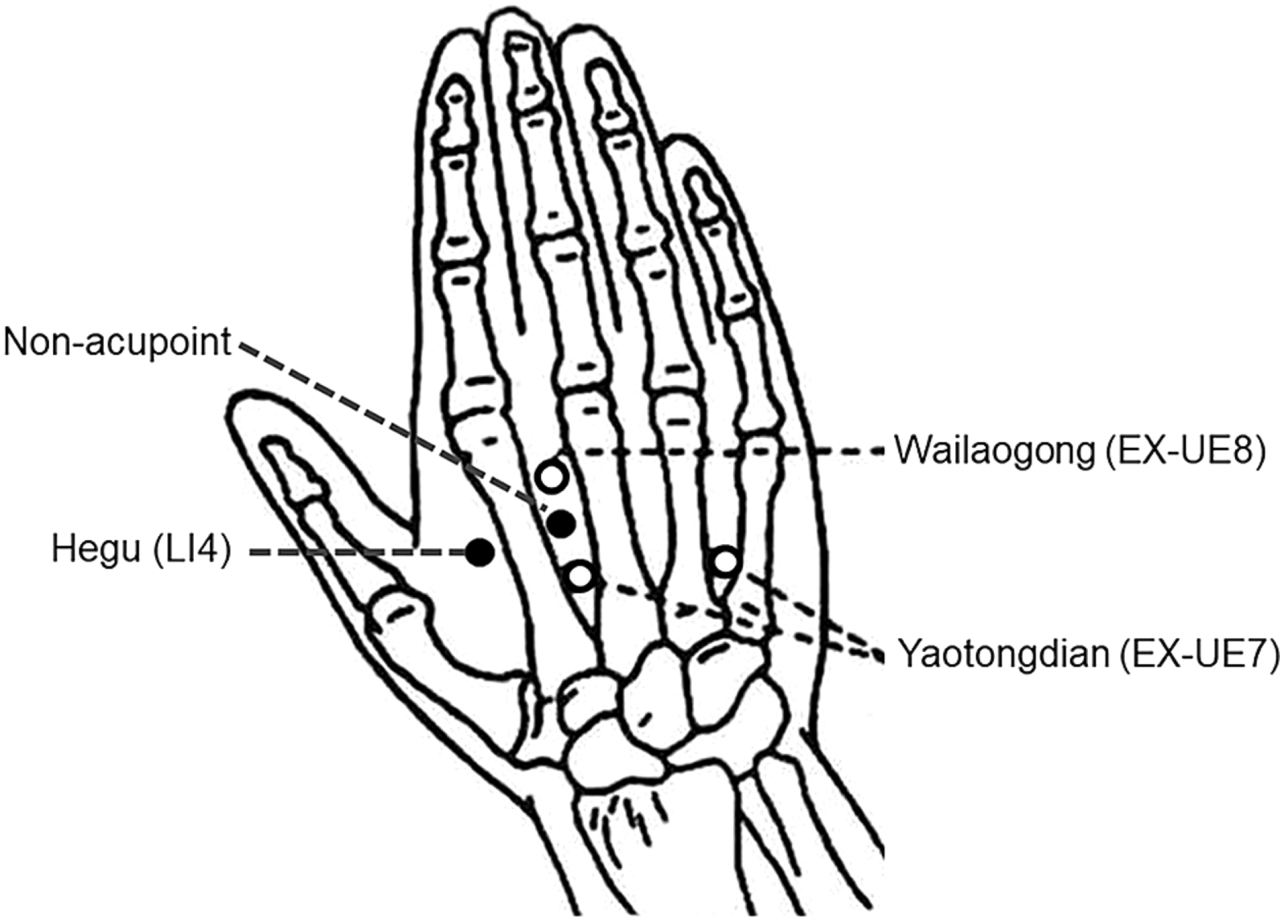
Anatomical localization of Hegu (LI4) and the “non-acupoint.”

“Hegu” (LI4) is located at the midpoint of the radial side of the 2nd metacarpal bone on the dorsal aspect of the hand. The “non-acupoint” is located between the 2nd and 3rd metacarpal bones on the dorsum of the hand, the midpoint between Wailaogong (EX-UE8) and Yaotongdian (EX-UE7) (Zaslawski et al., 2003) (Figure 2).

#### 2.4.2 Acupuncture methods

**Figure 3:**
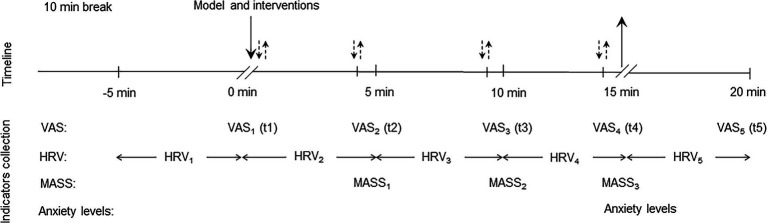
Timeline of the experimental procedure.↓: Needle insertion;↑: Needle withdrawal;↓↑: Reinforcement–reduction methods.

The participants were seated with their right hand exposed and extended into a cloth curtain to shield their vision. The application site was sterilized with 75% alcohol, and the intervention was carried out strictly according to the group intervention protocol by an administrator. Needling was not performed in the model group. In the sham acupuncture group, a tube needle (0.18 × 25 mm, 0.35 × 25 mm, Huatuo, Suzhou, China) was used to simulate needle insertion at a non-acupoint, touching the skin without breaking it. In the acupuncture group, the needle was inserted 0.5–0.8 inches into the Hegu point and left for 15 min. Reinforcement–reduction methods (uniform lifting and thrusting combined with twirling and rotating, 60 times/min) were performed for 30 s immediately, 4 min, 9 min, and 14 min after needle insertion (Figure 3).

### 2.5 Indicators

#### 2.5.1 Indicators of analgesic effects

(1) Pain level: Paper-based VAS assessment (Delgado et al., 2018). The test uses a 10-point intensity rating scale, where the participant is asked to rate their pain between “0,” indicating no pain, and “10,” indicating excruciating pain. VAS values were recorded at time intervals of 1 (t1), 5 (t2), 10 (t3), and 15 min after needling (t4), and again at 5 min after needle withdrawal (t5).

(2) Effective rate: According to the pain characteristics of the capsaicin patch model, one of VAS_3_, VAS_4_, and VAS_5_ ≥ VAS_1_ was selected as ineffective. Effective rate = (sample size - ineffective sample size)/sample size.

#### 2.5.2 Indicators of heart rate variability (HRV)

Short-term 5-min recordings were recorded by ECG (Li C. T. et al., 2022). The night before the test, the participants were asked to get adequate sleep, avoid strenuous exercise, and consume no alcohol, tea, or coffee. Following a 10-min break, the participants were seated, and ECG data were collected with a micro-ECG recorder R211B (Healink, Bengbu, China) for 5 min before modeling, 0–5 min, 5–10 min, 10–15 min, and 15–20 min after modeling. Excessive activity was avoided during collection, and the environment was kept quiet and at room temperature (24 ± 1°C). TP, LF, HF, and LF/HF of the frequency domain analysis and SDNN and RMSSD of the time domain analysis were selected to assess overall HRV, vagal activity, sympathetic activity, and sympathetic-vagal balance (Wu et al., 2023). Parameter analyses were performed using the Kubios HRV version 3.1.0 software (Kubios Oy: Kuopio, Finland).

#### 2.5.3 Indicators of sensory evaluation

At 5 min, 10 min, and the end of needling, the participants were asked whether they felt a needle sensation and its intensity, which was assessed using the Massachusetts General Hospital acupuncture sensation scale (MASS) (Li et al., 2017). The average of the three MASS scores was taken as the final intensity of the needle sensation. At the end of needling, the anxiety levels of the participants were measured using the same evaluation as the VAS (Kou et al., 2007).

### 2.6 Data processing

The data were entered independently by two research staff members. Statistical analyses were performed using IBM SPSS Statistics for Windows, version 23.0. An independent sample t-test was used for pre- and post-comparisons. The chi-square test and one-way analysis of variance (ANOVA) test were used for group comparisons. Repeated-measures ANOVA was used to analyze data from multiple measurements. Pearson’s correlation was used for correlation analysis. A value of *p* < 0.05 was considered a statistically significant difference.

## 3 Results

### 3.1 Participants and baseline

**Figure 4:**
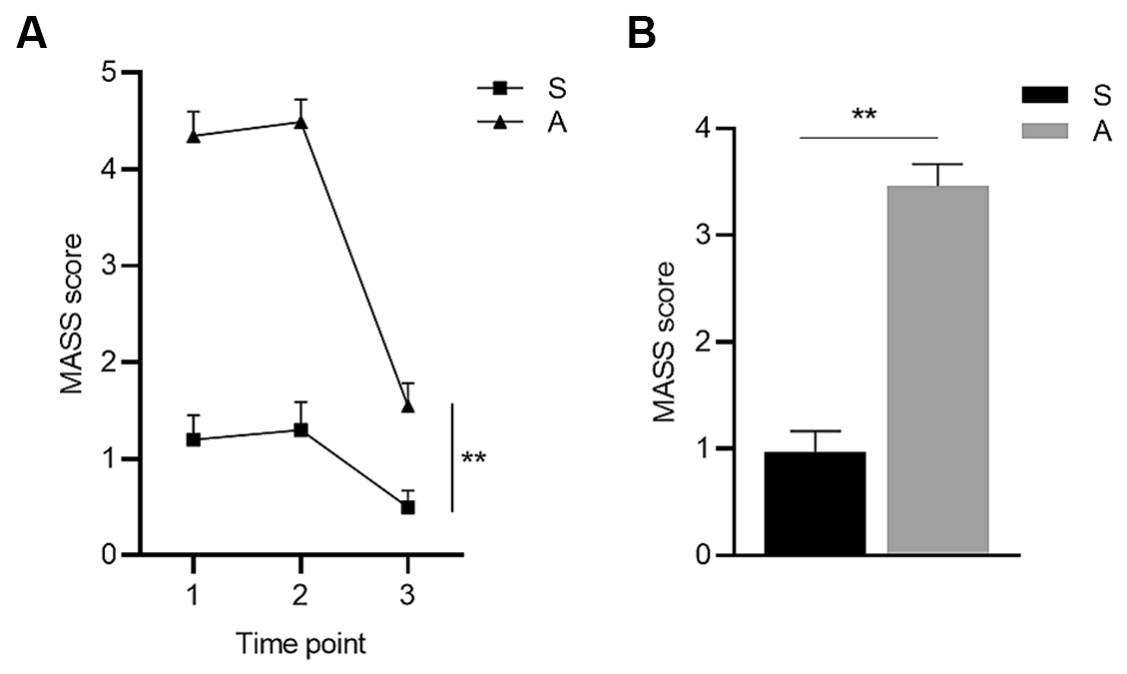
Comparison of MASS between groups. **(A)** shows the MASS curves of the sham acupuncture and the acupuncture group at three time points and **(B)** shows the comparison of average MASS scores between the two groups. ^*^ *p* < 0.05, ^**^ *p* < 0.01. Groups: S, sham acupuncture group, *n* = 31; A, acupuncture group, *n* = 61. The error bars indicate the standard errors. Time points 1–3: 5 min (t1), 10 min (t2), and the end of needling (t3).

There was no difference in age or sex between the groups (Table 1). The MASS curves and the average MASS score were significantly higher in the acupuncture group than in the sham acupuncture group, and the difference was statistically significant (*p* < 0.001, *p* < 0.001) (Figure 4).

**Table 1:** Basic characteristics of participants among groups.

	Model group (*N* = 30)	Sham acupuncture group (*N* = 31)	Acupuncture group (*N* = 61)	*p*
Sex				0.823
Female	22	22	41	
Male	8	9	20	
Age (year)	19.5 ± 0.7	19.5 ± 0.9	19.6 ± 0.8	0.744

Age is expressed as mean ± standard deviation.

### 3.2 Analgesic effect

**Figure 5:**
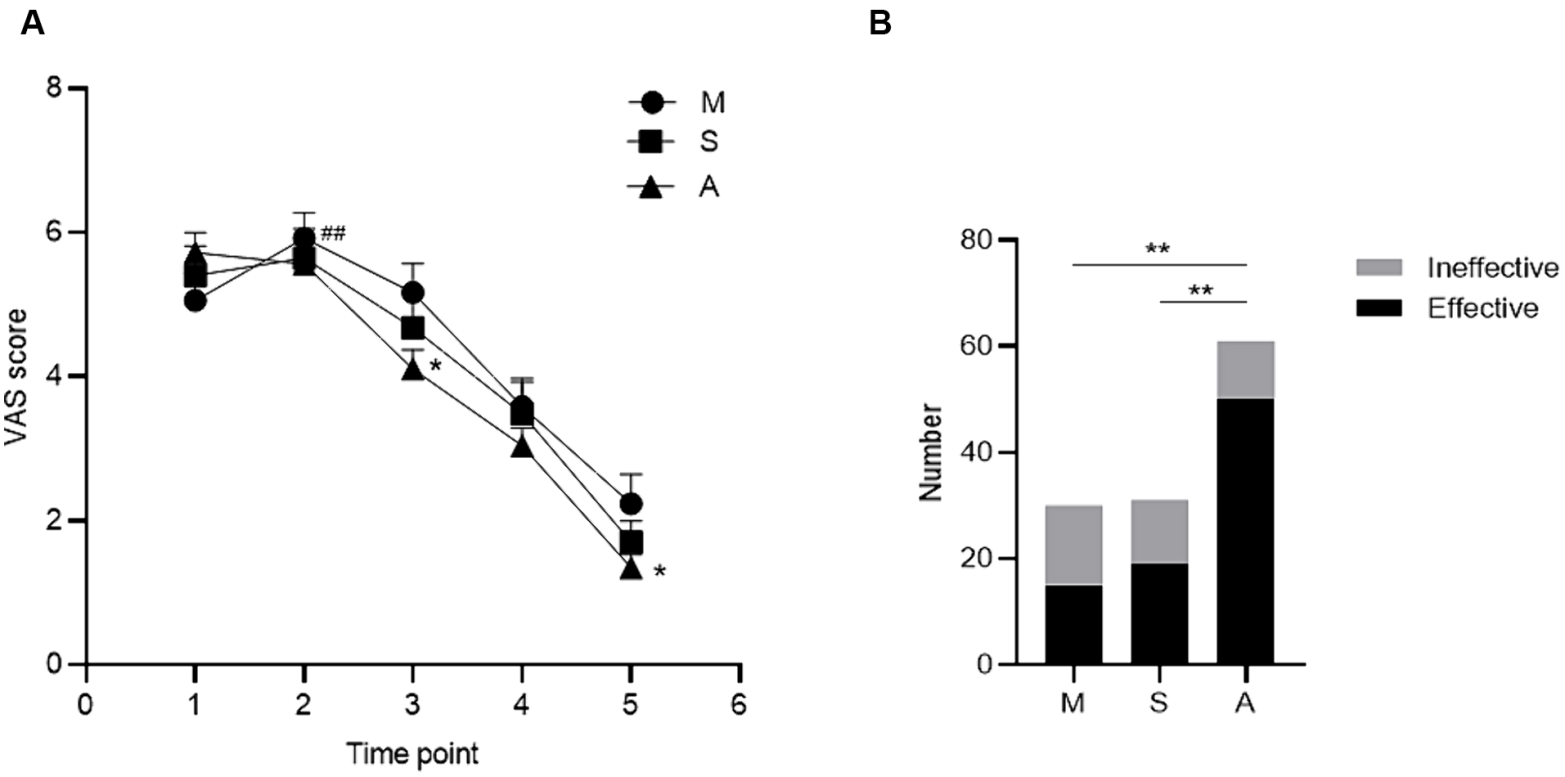
Comparison of analgesic effect among groups. **(A)** shows the VAS curves of the three groups at five time points, and **(B)** shows the comparison of the effective rates of the three groups. Compared with baseline, ^#^ *p* < 0.05, ^##^ *p* < 0.01; compared among groups, ^*^ *p* < 0.05, ^**^ *p* < 0.01. Groups: M, model group, *n* = 30; S, sham acupuncture group, *n* = 31; A, acupuncture group, *n* = 61. The error bars indicate the standard errors. VAS: visual analog scale. Time points 1–5: 1 (t1), 5 (t2), 10 (t3), and 15 min after needling (t4), and at 5 min after needle withdrawal (t5).

Compared with the baseline pain level (t1), the VAS score peaked after 5 min (t2) in the model group, and the difference was statistically significant (*p* = 0.005), while no differences were observed in the acupuncture and sham acupuncture groups (*p* = 0.465, *p* = 0.500) (Figure 5A).

Compared with that in the model group, the pain level decreased in the acupuncture group, and the difference in the VAS score was statistically significant at 10 min after needling (t3) (*p* = 0.038) and 5 min after needle withdrawal (t5) (*p* = 0.020). There were no significant differences in the sham acupuncture group (*p* = 0.402 and *p* = 0.221, respectively) (Figure 5A).

Comparing the total effective rate of each group, 82.0% in the acupuncture group was the highest, which was statistically significant compared with 50.0% in the model group (*p* < 0.001) and 61.3% in the sham acupuncture group (*p* < 0.001). No significant differences were observed between the model and sham acupuncture groups (*p* = 0.370) (Figure 5B).

### 3.3 Effect on heart rate variability (HRV)

#### 3.3.1 Frequency domain indicators

**Figure 6:**
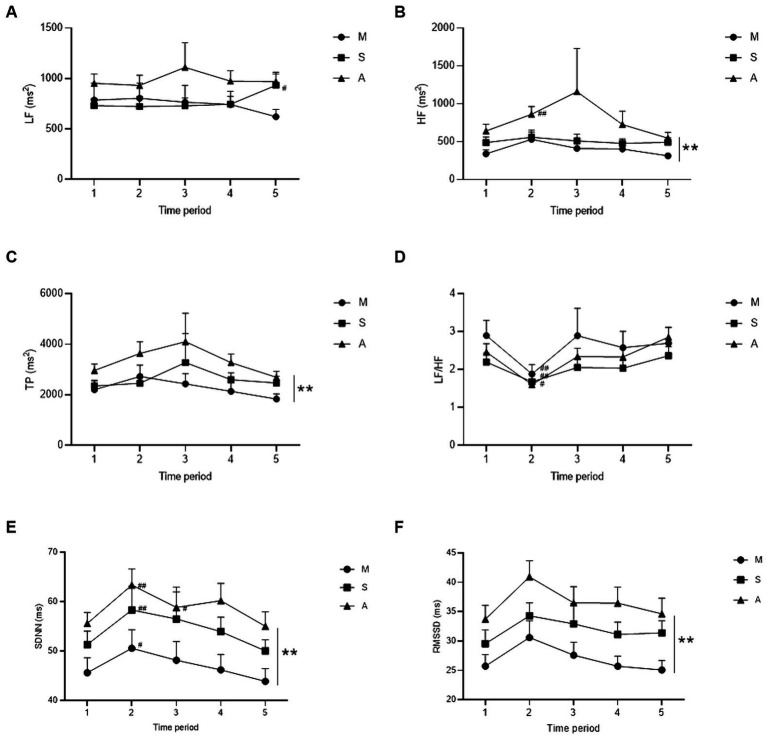
Comparison of HRV indicators for each group. **(A–D)** show the frequency domain indicators of LF, HF, TP, and LF/HF curves for the three groups at five time periods, respectively. **(E,F)** show the time domain indicators of SDNN and RMSSD curves for the three groups at five time periods, respectively. Compared with baseline, ^#^ *p* < 0.05, ^##^ *p* < 0.01; compared among groups, * *p* < 0.05, ** *p* < 0.01. Groups: M, model group, *n* = 30; S, sham acupuncture group, *n* = 31; A, acupuncture group, *n* = 61. The error bars indicate the standard errors. LF: low-frequency power, HF: high-frequency power, TP: total power, and LF/HF: ratio of LF to HF. SDNN: standard deviation of the normal-normal intervals, RMSSD: root mean square of successive interval differences. Time periods 1–5: 5 min before modeling (t1), 0–5 min (t2), 5–10 min (t3), 10–15 min (t4), and 15–20 min after modeling (t5).

Compared with that at baseline (t1), LF in the sham acupuncture group increased (*p* = 0.030) at the end of the intervention (t5) (Figure 6A), and HF in the acupuncture group increased (*p* = 0.005) from 0–5 min of the intervention (t2) (Figure 6B). During the same period, the LF/HF decreased in the model (*p* = 0.001), sham acupuncture (*p* = 0.048), and acupuncture groups (*p* < 0.001) (Figure 6D). No differences were observed in the frequency domain indicators for the remaining periods.

Compared with those in the model group, HF and TP levels increased in the acupuncture group, and the difference was statistically significant (*p* = 0.045, *p* = 0.041); no difference was observed in the sham acupuncture group (*p* = 0.632, *p* = 0.542) (Figures 6B,C). LF and LF/HF did not differ between the groups (model vs. sham, *p* = 0.857; model vs. acupuncture, *p* = 0.077; acupuncture vs. sham, *p* = 0.113) (Figures 6A,D).

#### 3.3.2 Time domain indicators

Compared with that at baseline (t1), SDNN increased in the model, sham acupuncture, and acupuncture groups (*p* = 0.018, *p* = 0.007, *p* = 0.007) from 0–5 min of the intervention (t2) (Figure 6E), and remained elevated in the acupuncture group (*p* = 0.034) from 5–10 min of the intervention (t3). No significant differences were observed in the remaining time intervals.

Compared with those in the model group, SDNN and RMSSD increased in the acupuncture group, and the difference was statistically significant (*p* = 0.002, *p* = 0.006); no difference was observed in the sham acupuncture group (SDNN *p* = 0.093, RMSSD *p* = 0.222) (Figures 6E,F).

### 3.4 Correlation analysis

#### 3.4.1 Massachusetts general hospital acupuncture sensation scale (MASS) and HRV

To understand the relationship between needle sensation and HRV, a correlation analysis bewteen average MASS scores and HRV indicators was performed in the acupuncture and sham acupuncture groups. The results revealed that the MASS was positively correlated with RMSSD_2_, LF_2_, RMSSD_4_, and TP_4_ (Table 2), and no correlation was observed with the other HRV indicators (data not shown).

**Table 2:** Significant correlations between MASS and HRV indicators.

		RMSSD_2_	LF_2_	RMSSD_4_	TP_4_
MASS	Pearson Correlation	0.229	0.207	0.227	0.217
	Sig.(2-tailed)	0.028	0.047	0.029	0.038
	N	92	92	92	92

RMSSD, root mean square of successive interval differences; LF, low-frequency power; TP, total power; MASS, Massachusetts General Hospital acupuncture sensation scale.

#### 3.4.2 MASS and visual analog scale (VAS)

To understand the relationship between needle sensation and acupuncture analgesia, a correlation analysis between average MASS score and VAS scores was performed in the acupuncture group. The results revealed that MASS was positively correlated with VAS_5_ (Table 3), and no correlation was observed at other time points (data not shown).

**Table 3:** Significant correlations between MASS and VAS.

		VAS_5_
MASS	Pearson Correlation	0.342
	Sig.(2-tailed)	0.007
	N	61

VAS, visual analog scale; MASS, Massachusetts General Hospital acupuncture sensation scale.

#### 3.4.3 MASS and anxiety levels

There was no significant difference in anxiety levels between the acupuncture and sham acupuncture groups (*p* = 0.926, data not shown). To understand the effect of needle sensation intensity on anxiety during acupuncture, a correlation analysis between average MASS score and anxiety levels was performed in the acupuncture group, and the results showed that they were positively correlated (Table 4).

**Table 4:** Correlation between MASS and anxiety levels.

		Anxiety levels
MASS	Pearson Correlation	0.433
	Sig.(2-tailed)	0.000
	N	61

MASS, Massachusetts General Hospital acupuncture sensation scale.

### 3.5 Adverse events

Each of the three groups experienced one case of excessive pain leading to intolerance, which was alleviated after halting the model and washing the affected skin. Two cases of mild fainting occurred in response to the needle in the acupuncture group, which were self-reported by the participants. It was attributed to excessive needle sensation and was resolved after stopping acupuncture and resting.

## 4 Discussions

This study found that AA was associated with increased vagal activity. The intensity of the “needle sensation” was positively correlated with vagal and sympathetic activity. A strong “needle sensation” may lead to higher levels of anxiety and weaken the sustained effects of AA.

In this study, a human pain model was prepared by applying capsaicin to the lower lip, which resulted in moderate-to-severe pain at 5 min and maintained mild-to-moderate pain for the next 15 min, indicating that the model was a success. Based on the pain location, we chose the Hegu (LI4) point, located on the back of the hand, as the needle acupoint. In Chinese medicine, the Hegu is connected to the face and mouth via the large intestine meridian, making it the preferred acupoint for treating facial and oral diseases (Qiao et al., 2020). From the VAS curves of the three groups, unlike the model and sham acupuncture groups, the acupuncture group experienced inhibition of the rising trend of pain and reduced pain at two subsequent time points. The efficiency in the acupuncture group was significantly higher than that in the other two groups, whereas there was no difference between the sham acupuncture and model groups. These results confirm the immediate analgesic effect of acupuncture, which is consistent with the results of several previous studies (Qiao et al., 2020; Liu et al., 2022).

We observed that HF, RMSSD, and SDNN, which reflected vagal activity, and TP, which reflected overall ANS activity, were elevated in the acupuncture group compared with those in the model group, and appeared concurrently with the analgesic effect within 5 min after acupuncture. There was no difference in the sham acupuncture group, nor was there any difference in LF, which reflected sympathetic activity. This suggests that AA is associated with increased vagal activity, which causes an increase in overall autonomic activity. Similar autonomic modulatory effects have been observed in clinical trials of acupuncture for migraine and lower back pain (Li Y. W. et al., 2022) and are consistent with the results of a recent meta-analysis (Hamvas et al., 2023). Auricular vagus nerve stimulation is an effective therapy for both chronic and acute pain (Likar et al., 2023). Studies have demonstrated that auricular acupuncture can also increase HRV and vagal activity during pain relief (Butt et al., 2020). These results suggest that the ANS is one of the targets of AA and that increasing vagal activity may be one of the mechanisms. A functional link exists between somatosensation and the ANS, and the activation of cutaneous sensory fibers can modulate disease conditions by affecting the autonomic nerves (Ma, 2020). In anti-inflammatory acupuncture studies, it was found that the dense neural distribution of myelinated Prokr2Cre fibers in the limbs may underlie the neural basis for the remote effects of limb acupoint stimulation in activating sympathetic and/or parasympathetic pathways (Ma, 2022). This study also provides insight into the autonomic regulatory basis of AA.

Functional connectivity in the brain is altered after acute pain stimulation. Neuroimaging studies have found that acupuncture enhances the post-stimulation spatial extent of the resting brain networks, including the hypothalamus (Lin and Chen, 2008), anterior cingulate cortex, periaqueductal grey matter, amygdala, hippocampus, middle temporal gyrus, and other brain regions that are resistant to nociception, memory, and emotion (Hauck et al., 2017). In addition, several minutes after the needle was removed, the connections formed by the default mode network with the hippocampus were enhanced by the acupuncture-induced sympathetic-to-parasympathetic transfer (Dhond et al., 2008), which may explain the continuous analgesic effect after needle withdrawal. Simultaneously, systems involved in autonomic control are strictly correlated with those involved in pain perception (Forte et al., 2022). These results confirm the role of the ANS in AA at the central level, and there may also be interactions between brain regions that allow for the integrated modulation of pain in the emotional and cognitive dimensions.

According to TCM and modern neurobiological theories, the intensity of acupuncture is an important factor influencing its effects (Ma, 2022). According to the TCM theory, needle sensation, also known as De Qi, is the sensation of soreness, numbness, heaviness, and distension experienced by participants during acupuncture, which can be transmitted through meridians and is considered the key to acupuncture efficacy. We found that needle sensation intensity was positively correlated with vagal, sympathetic, and overall autonomic activities, with the effects on vagal activity being stronger and lasting longer. Thicker myelin is associated with faster impulses, and electrophysiological recordings revealed that vagal reflexes can be driven by the activation of thinly myelinated Aδ fibers and can be further enhanced by activation of unmyelinated C fibers, whereas sympathetic reflexes may require C fiber activation (Ma, 2022). This may explain why the effects of needle sensation on the vagus nerve are more pronounced and long-lasting and why the pain score 5 min after needle withdrawal is positively correlated with needle sensation. Moderate needle sensation activates the vagus nerve to produce analgesia, whereas excessive needle sensation may activate sympathetic nerves, thereby antagonizing vagal activity and weakening the continuous effects of AA. Simultaneously, a strong needle sensation triggers higher anxiety levels, further activating sympathetic nerves (Lambert et al., 2010) and interfering with AA. Combined with the causes of needle-related fainting, this suggests that a stronger needle sensation is not better for patients during AA (Lee et al., 2014). Acupuncture is an integrative therapy that encompasses both physiological and psychological modulation. Choosing the appropriate intensity of acupuncture to achieve the best clinical outcome is worth considering. Whether HRV is an objective indicator of needle sensation requires further investigation.

Some studies have reported results contradictory to ours. One study concluded that AA was not associated with HRV (De Kooning et al., 2015). Another study found that acupuncture reduced sympathetic tone and improved vagal tone in fatigued states but not in non-fatigued states (Xiong et al., 2022). There are individual variations in HRV, and the autonomic function of an organism is related to many factors, such as age, sex, smoking, alcohol consumption, body position, respiration, and even level of education (Strüven et al., 2021), which may lead to different conclusions. The fact that acupuncture works only under pathological conditions may also be a factor (Lee et al., 2010).

This study has some limitations. First, the distribution of needle sensations was relatively concentrated; therefore, stratified analyses based on needle sensation intensity were not performed. More intuitive conclusions can be drawn by comparing the effects of different needle sensation intensities on HRV. Second, this study observed acute pain and did not examine chronic pain, which has a different mechanism and is more common in clinical settings.

In conclusion, AA was associated with enhanced vagal activity, the intensity of needle sensation was positively correlated with vagal and sympathetic activities, and excessive needle sensation may weaken the analgesic effects of acupuncture. Acupuncture is an effective means of regulating autonomic function, and needle sensation may be an important modulator, which provides a basis for an in-depth discussion of the autonomic mechanism of acupuncture. Therefore, how acupuncture can be used to treat autonomic dysfunction deserves further study.

## Data availability statement

The raw data supporting the conclusions of this article will be made available by the authors, without undue reservation.

## Ethics statement

The studies involving humans were approved by the Ethics Committee of Hunan University of Medicine. The studies were conducted in accordance with the local legislation and institutional requirements. The participants provided their written informed consent to participate in this study.

## Author contributions

ZeL: Writing – original draft, Investigation. JH: Writing – original draft, Investigation. DY: Writing – original draft, Investigation. SL: Writing – original draft, Investigation. SZ: Writing – original draft, Data curation, Investigation. MZ: Writing – original draft, Data curation, Investigation. ZhL: Writing – original draft, Investigation. CJ: Writing – original draft, Funding acquisition, Investigation. XY: Writing – original draft, Investigation. YZ: Writing – review & editing, Formal Analysis, Methodology. TH: Writing – review & editing, Visualization, Formal Analysis, Methodology. MF: Funding acquisition, Writing – review & editing, Project administration, Conceptualization, Methodology.
